# Preliminary Validity and Reliability Evidence for a Derived 12-Item Greek Short-Form Model of the Quality Indicators for Physiotherapy Management of Hip and Knee Osteoarthritis (QUIPA)

**DOI:** 10.3390/healthcare14162464

**Published:** 2026-08-10

**Authors:** Christina Gkaripi, Maria Moutzouri, Eirini Grammatopoulou, Petros Tatsios, Stefanos Karanasios, Georgios Krekoukias

**Affiliations:** 1 Department of Physiotherapy, University of West Attica, 12243 Athens, Greece; 2 Laboratory of Advanced Physiotherapy, Department of Physiotherapy, University of West Attica, 12243 Athens, Greece; moutzouri@uniwa.gr (M.M.); igrammat@uniwa.gr (E.G.); ptatsios@uniwa.gr (P.T.); skaranasios@uniwa.gr (S.K.); gkrekoukias@uniwa.gr (G.K.)

**Keywords:** cross-cultural adaptation, osteoarthritis, physiotherapy, quality indicators, process-of-care measure, structural validity, reliability

## Abstract

**Highlights:**

**What are the main findings?**

- The derived 12-item Greek QUIPA model showed preliminary validity and reliability evidence in adults with hip and/or knee osteoarthritis receiving physiotherapy in private clinics in Attica.
- Item-level test–retest agreement should be interpreted using categorical reliability indices, while the total proportion-positive score is currently more defensible than factor-level scores because internal consistency was borderline for the total score and poor for some provisional factors.

**What are the implications of the main findings?**

- The Greek QUIPA may support a descriptive audit of patient-reported physiotherapy care processes in settings similar to those studied.
- Further validation is required in larger and more diverse samples, including confirmatory factor analysis, convergent validity testing, and assessment in public-sector and community settings.

**Abstract:**

Background/Objectives: Osteoarthritis of the hip and/or knee is a major contributor to pain, disability, and reduced quality of life. The aim of this study was to provide preliminary validity and reliability evidence for a Greek adaptation of the Quality Indicators for Physiotherapy Management of Hip and Knee Osteoarthritis (QUIPA) questionnaire. Methods: This cross-sectional methodological study involved cross-cultural adaptation and psychometric evaluation of the Greek QUIPA in 90 adults aged 45–65 years with hip and/or knee osteoarthritis attending private physiotherapy clinics. Participants completed the questionnaire twice, seven days apart. Structural validity was explored using exploratory factor analysis, and exploratory subgroup analyses by age and sex were also conducted. Internal consistency, categorical item-level test–retest reliability, total-score test–retest reliability, and floor/ceiling effects were assessed. Results: Exploratory factor analysis of the initial 18-item pool suggested a three-factor 12-item solution explaining 51.5% of the total variance, with eigenvalues of 3.11, 1.58, and 1.49. Internal consistency was borderline for the total score (Cronbach’s alpha = 0.675) and the first factor (alpha = 0.662), and poor for the second (alpha = 0.424) and third (alpha = 0.598) factors. Item-level observed agreement ranged from 79.7% to 97.8%, with Cohen’s kappa values ranging from 0.580 to 0.887. Total-score test–retest reliability was good (ICC = 0.829, 95% CI = 0.748–0.896), although the total proportion-positive score increased by 5.6 percentage points at retest. Ceiling effects were observed for Q1, Q7, and Q8. Conclusions: The present study provides preliminary validity and reliability evidence for a derived 12-item Greek QUIPA short-form model. However, given the marginal sampling adequacy, low-to-moderate communalities, six-item reduction from the original 18-item questionnaire, categorical item properties, and borderline-to-poor internal consistency of some factors, the findings should be interpreted cautiously. At present, the Greek QUIPA is best interpreted using the total proportion-positive score, whereas factor-level scores should be treated as exploratory. Independent confirmatory factor analysis and further validation in larger and more diverse samples are required.

## 1. Introduction

Osteoarthritis (OA) is a major cause of chronic pain and disability worldwide, most commonly affecting the hip and knee joints [1,2]. Its rising prevalence, driven by population ageing and modifiable risk factors, places a substantial burden on both healthcare systems and society [2,3]. Beyond structural joint changes, OA has a profound impact on physical function, psychological well-being, and overall quality of life [1,4].

Physiotherapy represents a cornerstone of conservative OA management, with international clinical guidelines consistently recommending exercise therapy, patient education, and support for self-management, alongside other interventions where appropriate [5,6,7]. Nevertheless, despite the availability of clear evidence-based recommendations, their implementation in routine clinical practice remains inconsistent, with considerable variability in treatment delivery and adherence across settings and practitioners [8,9].

This variability highlights the need for robust tools to assess the quality of physiotherapy management [8,10]. In this context, it is important to distinguish patient-reported outcome measures from patient-reported process-of-care measures. Patient-reported outcome measures (PROMs), such as the Western Ontario and McMaster Universities Osteoarthritis Index (WOMAC), the Knee Injury and Osteoarthritis Outcome Score (KOOS), and the Hip Disability and Osteoarthritis Outcome Score (HOOS), assess symptoms, pain, and functional status. By contrast, process-of-care measures evaluate whether recommended components of care were delivered. PROMs therefore do not capture whether physiotherapy care is provided in accordance with evidence-based recommendations [8,11].

To address this gap, the Quality Indicators for Physiotherapy Management of Hip and Knee Osteoarthritis (QUIPA) questionnaire was developed as a patient-reported measure of whether key evidence-based components of physiotherapy care were delivered. The original English QUIPA comprised 18 items organized into three content-based subscales and demonstrated acceptable subscale/total test–retest reliability and hypothesis-based construct validity, but criterion validity was inadequate and further refinement before implementation was recommended [12]. More broadly, guideline-derived healthcare quality indicators for physiotherapy management of hip and knee osteoarthritis were developed using a Delphi process [13]. A subsequent Turkish adaptation retained the full 18-item instrument and reported favorable reliability and an exploratory three-factor solution [14].

Despite its clinical importance, the QUIPA questionnaire has not yet been psychometrically examined in Greek-speaking patients with hip and/or knee osteoarthritis. This represents a clear gap within the Greek healthcare context, as it limits the ability of physiotherapists to systematically evaluate the quality of care provided to individuals with OA. Cross-cultural adaptation and preliminary psychometric evaluation are therefore essential to bridge this gap, ensuring conceptual equivalence and measurement reliability in the Greek linguistic and cultural setting [15,16].

To address this need, the aim of the present study was to provide preliminary validity and reliability evidence for the QUIPA questionnaire in Greek-speaking adults with hip and/or knee osteoarthritis.

## 2. Materials and Methods

### 2.1. Study Design

The present cross-sectional methodological study aimed to provide preliminary validity and reliability evidence for the QUIPA questionnaire in Greek-speaking adults with hip and/or knee osteoarthritis.

### 2.2. Participants

A total of 90 adults aged 45–65 years were enrolled through consecutive sampling from 10 private physiotherapy clinics selected to ensure geographical representation across the Attica region, Greece. Inclusion required a prior diagnosis of hip and/or knee osteoarthritis established by an orthopaedic physician based on clinical evaluation, supported by imaging findings where available (e.g., X-ray, MRI, or CT). All participants had been referred to physiotherapy and were receiving treatment at the time of recruitment.

Eligibility was confirmed by physiotherapists at each facility through review of medical records and referral documentation available at the time of enrollment. Patients were excluded if they had a history of total joint arthroplasty, inflammatory arthritis, neurological or psychiatric disorders, active malignancy, or inability to provide informed consent. Patients with end-stage osteoarthritis requiring or scheduled for surgical intervention were also excluded. Severe comorbidities, as well as substance or alcohol dependence, were additional exclusion criteria. Substance and alcohol dependence were assessed based on medical records and participant self-report at the time of recruitment. Radiographic severity grading (e.g., Kellgren–Lawrence classification) was not systematically available and was therefore not included as a criterion.

The sample size (*n* = 90) was determined based on a ratio of five participants per item (rule of thumb) [16,17]. To support the sampling adequacy, Kaiser–Meyer–Olkin (KMO) index and Bartlett’s test of sphericity were evaluated [17,18].

### 2.3. Instrument

The Quality Indicators for Physiotherapy Management of Hip and Knee Osteoarthritis (QUIPA) questionnaire was originally developed by Pek Ling Teo and colleagues in 2020 to evaluate the quality of physiotherapy care provided to individuals with hip and knee osteoarthritis in accordance with evidence-based recommendations [12]. Unlike traditional patient-reported outcome measures that primarily assess pain, symptoms, or functional limitations, the QUIPA focuses on the process and content of physiotherapy management, including patient education, exercise prescription, self-management support, individualized assessment, and referral practices.

The original QUIPA consists of 18 items representing key quality indicators of physiotherapy management for hip and knee osteoarthritis. Most items are answered using “Yes/No” response options, while a “Don’t remember” option is provided for specific items. The questionnaire instructions specify a 12-week recall period, require respondents to select only one response per item, and include skip rules for selected items: if Item 10 is answered “No”, Item 12 is omitted; if Item 13a is answered “No” or “Don’t remember”, Item 13b is omitted. The total score is calculated as the percentage of “Yes” responses over the total number of “Yes” and “No” responses, with higher scores indicating greater adherence to evidence-based physiotherapy recommendations.

The original English QUIPA demonstrated acceptable subscale/total test–retest reliability and hypothesis-based construct validity, but criterion validity was inadequate and the authors recommended further refinement before implementation [12]. The Turkish adaptation later retained the original 18-item structure and reported stronger internal consistency and an exploratory three-factor solution [14]. However, before the present study, no Greek QUIPA model had been psychometrically examined in Greek-speaking adults with hip and/or knee osteoarthritis.

The QUIPA questionnaire was used in the present study under the Creative Commons Attribution 4.0 International License [12]. In addition to the questionnaire responses, demographic and clinical variables collected in the present study included age, sex, body weight, height, body mass index (BMI), disease duration, and affected joint diagnosis/site, classified as hip, knee, or both.

### 2.4. Scoring and Handling of QUIPA Responses

The Greek-translated QUIPA was administered in its full 18-item form. Responses were coded according to the scoring logic of the original QUIPA tool. ‘Yes’ responses were considered positive indicators of received evidence-based physiotherapy care, whereas ‘No’ responses were considered negative indicators. ‘Don’t remember’, missing, and not applicable responses were excluded from the denominator. Proportion-positive scores were calculated as the number of positive responses divided by the number of valid ‘Yes’ and ‘No’ responses, multiplied by 100. Higher scores indicate greater patient-reported adherence to evidence-based physiotherapy care processes. The same response-coding approach was applied to the retained item set after exploratory item refinement.

For exploratory factor-level scores, the same proportion-positive logic may be applied within each provisional factor, but such scores should be interpreted cautiously because structural validity and internal consistency remain preliminary. Because Q12 and Q13b are conditionally non-applicable in the original instrument, their original conditional response logic should be retained in the scoring denominator. At present, the total proportion-positive score should be preferred for descriptive interpretation, whereas factor-level scores should be treated as exploratory.

### 2.5. Procedures

Data collection was conducted in two phases. At baseline, participants completed the Greek-translated QUIPA questionnaire during their physiotherapy sessions. Demographic and clinical data (age, sex, BMI, disease duration, and affected joint) were recorded. To assess test–retest reliability, the questionnaire was re-administered after seven (7) days under consistent conditions within the same clinical setting, with no planned changes in treatment during this interval.

### 2.6. Cross-Cultural Adaptation

The original QUIPA questionnaire was used under a Creative Commons Attribution 4.0 International License [12]. The cross-cultural adaptation process followed a five-stage procedure: forward translation, synthesis of the translated versions, back-translation, expert-panel review, and pilot testing.

The six-member expert panel consisted of two physiotherapists and co-authors who also served as forward translators (G.K. and C.G.), two bilingual native English-speaking back-translators, an English-language specialist, and a methodologist (E.G.). G.K. is an Assistant Professor in the Department of Physiotherapy at the University of West Attica, with 28 years of professional experience as a physiotherapist, including 24 years in manual therapy, and 22 years of teaching experience in higher education. He is proficient in both Greek and English, having spent several years in England. C.G., currently a doctoral candidate in the same department, was a master’s student at the time of the translation and review process. She had at least five years of professional experience in physiotherapy clinics, rehabilitation centres, and home-based physiotherapy, working with both musculoskeletal and neurological cases, and held a B2-level qualification in English.

G.K. and C.G. independently translated the original QUIPA questionnaire from English into Greek. The two Greek translations were then compared item by item, and differences in wording were discussed until consensus was reached on a single reconciled Greek version. This version was independently back-translated into English by two bilingual native English speakers who were fluent in Greek, had no clinical background or prior knowledge of the QUIPA questionnaire, and had not been given access to the original English version. Each back-translator produced a separate English version.

The English-language specialist, who held a degree in English Language and Literature and had 10 years of professional experience in English-language teaching and linguistic review, examined the translated materials for linguistic accuracy, clarity, and fidelity to the original questionnaire. The methodologist (E.G.), a physiotherapist and university professor with more than 35 years of professional experience, reviewed the methodological aspects of the adaptation process and contributed to the assessment of semantic, idiomatic, experiential, and conceptual equivalence across versions.

The full expert panel jointly reviewed the original questionnaire, the two forward translations, the reconciled Greek version, and the two back-translations. Any differences or inconsistencies identified during this process were discussed until consensus was reached, with particular attention to semantic, idiomatic, experiential, and conceptual equivalence.

The resulting pre-final Greek version was pilot-tested by C.G. in 10 patients with hip and/or knee osteoarthritis. A brief cognitive debriefing procedure was used to examine the clarity and comprehensibility of each item. Participants were asked whether each item was clear and easy to understand, whether any words or expressions were confusing or ambiguous, and what they understood each item to mean. No issues requiring revision were identified; therefore, no further modifications were made. No formal quantitative assessment of item-level content validity, such as a content validity index, was undertaken.

### 2.7. Ethical Considerations

Ethical approval was obtained from the University of West Attica ethics committee (approval number: 102378; date of approval: 4 November 2024). All participants provided written informed consent prior to participation. The study adhered to the Declaration of Helsinki [19] and applicable data protection regulations [20]. All data were anonymized.

### 2.8. Statistical Analysis

Statistical analyses were performed using IBM SPSS Statistics version 29.0, with the level of significance set at α = 0.05. The Kaiser–Meyer–Olkin (KMO) index and Bartlett’s test of sphericity were calculated to assess the suitability of the data for factor analysis [18]. Descriptive statistics were used to summarize the total proportion-positive score and item-level response distributions. For scoring and reliability analyses, ‘Yes’ and ‘No’ responses were treated as valid responses, whereas ‘Don’t remember’, missing, and not applicable responses were excluded from the relevant denominator. For the exploratory factor analysis and internal consistency analyses, non-substantive responses were treated as missing.

Structural validity was explored using exploratory factor analysis (EFA) of the initial 18-item pool through Principal Axis Factoring with Oblimin rotation. This approach was retained as an exploratory analysis, but the categorical/process-indicator nature of the QUIPA items and the presence of sparse response categories were considered when interpreting the results. Factor retention was based on: (a) eigenvalues > 1.0, (b) inspection of the scree plot, (c) explained variance, and (d) conceptual interpretability [17,18]. Items with factor loadings ≥ 0.40 were considered salient. Communalities were inspected descriptively; values below 0.40 were interpreted as indicating limited shared variance and were considered alongside loading patterns, cross-loadings, reliability indices, and conceptual coherence, rather than as an automatic exclusion criterion. Exploratory subgroup analyses by age and sex were also undertaken, but these were not specified as formal a priori known-groups hypotheses. Internal consistency was evaluated using Cronbach’s alpha coefficient, with values around 0.70 commonly used as a rule-of-thumb for acceptable consistency in preliminary research [21]. Item-level test–retest reliability was assessed using observed percentage agreement and Cohen’s kappa because the retained QUIPA items are categorical. Total-score test–retest reliability was assessed using the intraclass correlation coefficient (ICC) based on a two-way mixed-effects, absolute-agreement, single-measures model, with values interpreted as poor (<0.50), moderate (0.50–0.75), good (0.75–0.90), and excellent (>0.90) [22]. A paired t-test was used to examine systematic change in the total proportion-positive score between baseline and retest. Floor or ceiling effects were defined as present when more than 85% of valid responses were negative or positive, respectively.

## 3. Results

A total of 90 participants were included in the study (60 women, 66.7%; 30 men, 33.3%), with a mean age of 57.4 years (SD = 6.7) and a mean disease duration of 3.7 years. The mean BMI was 29.13 kg/m^2^. Disease localization included the knee (58.9%), hip (32.2%), and both joints (8.9%). Detailed demographic and clinical characteristics are presented in Table 1.

The mean total proportion-positive score for the 12-item Greek QUIPA model was 68.3% (SD = 22.4), with scores ranging from 0% to 100%.

Prior to exploratory factor analysis, sampling adequacy was assessed using the Kaiser-Meyer-Olkin (KMO) measure and Bartlett’s test of sphericity [17,18]. Before item reduction, the initial 18-item pool showed marginal sampling adequacy (KMO = 0.593), indicating that the factor analytic findings should be interpreted cautiously [18]. Bartlett’s test of sphericity was significant, χ^2^(153) = 205.59, *p* = 0.003, indicating sufficient inter-item correlations to proceed with exploratory factor analysis [17,18].

The distributional characteristics of the initial 18-item pool were assessed using skewness and kurtosis [18]. Some items showed substantial deviation from normality, with skewness ranging from −0.12 to 5.29 and kurtosis from −2.07 to 26.55. Given the categorical/process-indicator nature of QUIPA items, these deviations were expected for indicators with high or low endorsement rates. Therefore, factor analysis was performed using Principal Axis Factoring, which is less dependent on multivariate normality than maximum likelihood extraction [17,18]. Nevertheless, the observed distributional asymmetry should be considered when interpreting the stability of the factor solution [17,18].

### 3.1. Structural Validity and Exploratory Subgroup Analyses

#### 3.1.1. Exploratory Factor Analysis (Principal Axis Factoring)

Exploratory factor analysis of the initial 18-item Greek translation suggested a three-factor 12-item solution explaining 51.5% of the total variance. Six items were removed because of inconsistent loading patterns, substantial cross-loadings, poor temporal stability, or limited conceptual coherence [16,23]. Specifically, Items 2 and 3 showed conceptually ambiguous and inconsistent loading patterns; Items 5, 10, and 17 showed substantial cross-loadings; and Item 6 showed unacceptable temporal stability, including a negative ICC. Factor loadings and communalities for the retained items are presented in Table 2. Detailed information on item retention, original-domain mapping, factor loadings, and communalities is provided in Supplementary Table S1A, while baseline response distributions, missingness, and categorical test–retest reliability indices for the original 18-item pool are provided in Supplementary Table S1B.

The retained solution should be interpreted cautiously because communalities were low-to-moderate (0.269–0.448). Only loadings ≥ 0.40 are displayed in Table 2; lower non-primary loadings were suppressed to improve readability. Correlations among provisional factor-level proportion-positive scores were low-to-moderate (r = 0.32–0.34), supporting the use of an oblique rotation while reinforcing that the factors should be interpreted as exploratory. The total proportion-positive score for the retained 12-item Greek QUIPA model was then calculated using the same scoring logic as the original QUIPA: positive responses divided by valid ‘Yes’ and ‘No’ responses, multiplied by 100.

**Figure 1:**
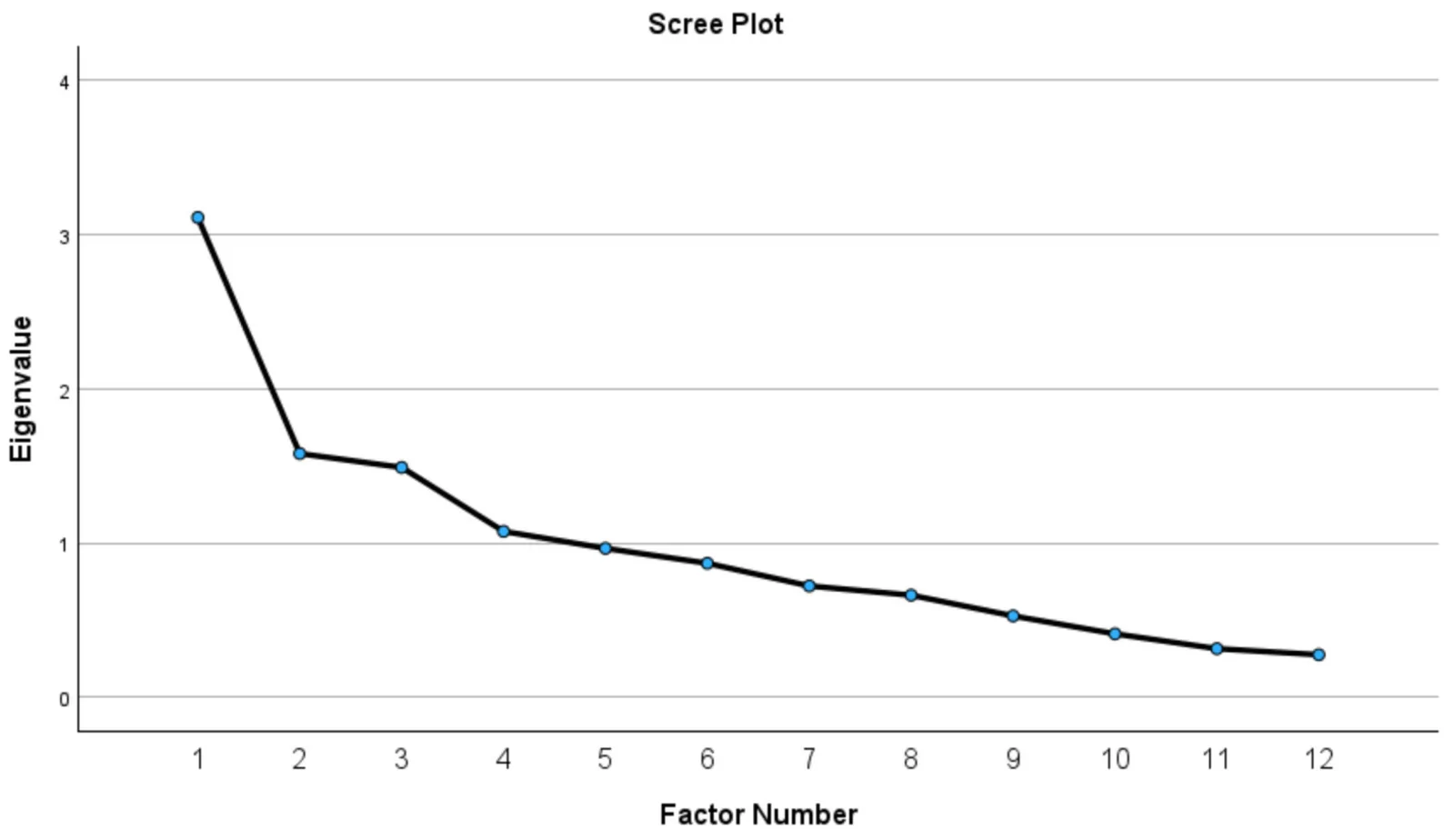
Scree plot of eigenvalues.

The scree plot showed an inflection after the third factor, supporting retention of a three-factor model. This was consistent with the eigenvalue > 1 criterion, as the three retained factors had eigenvalues of 3.11, 1.58, and 1.49, respectively, explaining 25.9%, 13.2%, and 12.4% of variance (51.5% cumulatively) (Figure 1).

#### 3.1.2. Exploratory Subgroup Analyses

The total proportion-positive score of the 12-item Greek QUIPA model was compared across three age groups: 45–50 years (M = 66.0%, SD = 26.3), 51–60 years (M = 69.4%, SD = 21.1), and 61–65 years (M = 68.2%, SD = 22.2). No statistically significant differences were observed across age groups, F(2,87) = 0.14, *p* = 0.871. Levene’s test indicated homogeneity of variances, *p* = 0.836.

An independent samples t-test was performed to compare the total proportion-positive score of the 12-item Greek QUIPA model between males and females. No statistically significant difference was observed between males (M = 70.1%, SD = 19.4) and females (M = 67.4%, SD = 23.8), t(88) = 0.55, *p* = 0.582. Levene’s test for equality of variances was non-significant, *p* = 0.295.

### 3.2. Reliability

The internal consistency of the 12-item three-factor QUIPA model was borderline for the total score (Cronbach’s α = 0.675) and for the first factor (α = 0.662). Internal consistency was poor for the second factor (α = 0.424) and the third factor (α = 0.598), indicating that factor-level scores should be interpreted cautiously. The results are summarized in Table 3.

Item-level test–retest reliability was evaluated using observed agreement and Cohen’s kappa because the retained QUIPA items are categorical. Observed agreement ranged from 79.7% to 97.8%, and Cohen’s kappa values ranged from 0.580 to 0.887 (Table 4), indicating moderate to almost perfect agreement. The lower kappa values for Q7, Q8, and Q12 should be interpreted alongside the high observed agreement and the ceiling or conditional response patterns for these items. At the score level, the total proportion-positive score showed good test–retest reliability using a two-way mixed-effects, absolute-agreement, single-measures ICC (ICC = 0.829, 95% CI = 0.748–0.896). However, the mean total proportion-positive score increased from 68.3% (SD = 22.4) at baseline to 73.9% (SD = 18.4) at retest, indicating a mean increase of 5.6 percentage points over seven days (95% CI = 3.3–7.9; *p* < 0.001).

### 3.3. Floor and Ceiling Effects

Item-level response distributions were inspected to evaluate potential floor and ceiling effects. Ceiling or floor effects were defined as present when more than 85% of valid responses were positive or negative, respectively. Valid positive percentages were calculated using only valid ‘Yes’ and ‘No’ responses in the denominator; ‘Don’t remember’, missing, and not applicable responses were excluded. Ceiling effects were observed for Q1, Q7, and Q8, with positive response rates of 95.6%, 92.2%, and 90.0%, respectively. No item demonstrated a floor effect. Q13a showed a high proportion of ‘Don’t remember’ responses (37.8%), suggesting possible recall difficulty for weight-management discussions, whereas Q13b had a high proportion of missing/not applicable responses (43.3%) because of the original conditional skip pattern. The results are summarized in Table 5.

## 4. Discussion

The present study provides preliminary validity and reliability evidence for a derived 12-item Greek QUIPA short-form model in adults with hip and/or knee osteoarthritis receiving physiotherapy in private clinics. Overall, the findings suggest that the instrument may be useful for describing patient-reported receipt of selected evidence-based physiotherapy care processes. However, the evidence should be interpreted cautiously because the retained model was derived from, rather than structurally equivalent to, the original 18-item QUIPA.

Exploratory factor analysis identified a three-factor model, supporting a multidimensional structure. However, the resulting factor composition does not reproduce the original QUIPA subscales exactly. Rather, it reorganizes retained items from the original Assessment and Management Planning, Core Recommended Treatments, and Adjunctive Treatments domains into a shorter Greek configuration. The structure should therefore be interpreted as an adapted, preliminary solution rather than as a direct structural replication of the original instrument, and confirmatory factor analysis in an independent sample is required.

These findings are only partly comparable with previous studies. The original QUIPA paper was designed around three content-based subscales and demonstrated acceptable subscale/total test–retest reliability and hypothesis-based construct validity, but it did not establish the present Greek factor structure and concluded that further refinement was needed before implementation [12]. The Turkish adaptation retained the full 18-item instrument and reported stronger total reliability and an exploratory three-factor solution [14]. Therefore, the current findings should not be interpreted as confirmation of structural stability across cultures.

In this context, the QUIPA differs fundamentally from commonly used patient-reported outcome measures in osteoarthritis, which primarily assess symptoms and functional status. In contrast, QUIPA evaluates whether physiotherapy care is delivered in accordance with evidence-based recommendations, including patient education, support for self-management, and individualized treatment planning. This distinction highlights its added value as a patient-reported measure of care quality rather than clinical outcome alone [24,25].

In the present study, item reduction from 18 to 12 items was necessary due to poor factor loadings, cross-loadings, poor reliability, and conceptual inconsistencies [16,23].

The present findings support a derived 12-item Greek QUIPA short-form model rather than a direct structural replication of the original 18-item instrument. Six items (Q2, Q3, Q5, Q6, Q10, and Q17) were removed because of inconsistent loading patterns, substantial cross-loadings, poor temporal stability, and limited conceptual coherence within the emerging factor solution. Although the retained items preserve broad representation of the original content areas, the removal of comorbidity assessment, psychosocial screening, management planning, review timing, specific exercise prescription, and footwear advice may have narrowed content coverage. This potential loss of content is particularly relevant to exercise prescription, which is a key component of conservative hip and knee osteoarthritis management and should be individualized to patient characteristics [26].

The provisional first factor (Q4, Q13a, Q13b, Q14, Q15) mainly reflects referral, education, and adjunctive support; the second factor (Q7, Q8, Q12) reflects information provision and exercise-adherence discussion; and the third factor (Q1, Q9, Q11, Q16) reflects assessment, treatment discussion, and individualized planning. These labels should be considered descriptive because the factor structure remains preliminary.

Because Q12 concerns adherence to a specific exercise programme but Q10 was removed, the current evidence supports a retained-item scoring model derived from the full Greek translation rather than a fully operational stand-alone short form. A fully stand-alone short-form version would require one of three options: retention of Q10 as a routing item, removal or rewriting of Q12, or development of revised retained-item instructions followed by cognitive debriefing and pilot testing of the conditional wording for Q12 and Q13b.

No statistically significant differences were observed across age groups or between males and females. These findings suggest that 12-item QUIPA scores did not differ by these demographic characteristics in the present sample.

However, because these analyses were exploratory and the sample size was limited, the absence of statistically significant differences should not be interpreted as evidence of measurement invariance or absence of bias. Future studies with larger samples should examine whether the Greek QUIPA performs similarly across demographic and clinical subgroups.

Regarding internal consistency, the 12-item model demonstrated a borderline Cronbach’s α for the total scale and poor internal consistency for the second and third factors. The borderline total alpha and the lower factor-level values suggest that the retained items should not be assumed to represent highly homogeneous latent constructs. This may partly reflect the process-indicator nature of QUIPA [12], where items capture distinct but related aspects of physiotherapy care delivery rather than interchangeable manifestations of a single underlying construct. Given the borderline internal consistency of the total score and poor internal consistency of some factors, the retained 12-item Greek QUIPA model should currently be interpreted primarily using the total proportion-positive score. Factor-level scores should be considered exploratory and are not recommended for applied interpretation until the structure is confirmed in an independent sample.

In terms of temporal stability, item-level categorical reliability indicated moderate to almost perfect agreement for the retained items. However, the mean total proportion-positive score was modestly higher at retest than at baseline, suggesting possible recall, learning, response-shift effects, or actual additional care received during the seven-day interval. This finding weakens a simple interpretation of temporal stability and should be considered a substantive psychometric limitation rather than only a minor statistical observation. For this reason, total-score stability should be re-examined in future studies with a pre-specified reliability analysis and a retest interval in which additional treatment exposure is carefully controlled.

Several limitations should be considered. First, although the sample met minimal rule-of-thumb requirements for exploratory factor analysis, sampling adequacy remained only marginal and several communalities were low-to-moderate, so the factor solution should be regarded as preliminary. Second, the factor analysis was conducted using an exploratory standard-correlation approach despite the categorical/process-indicator nature of the QUIPA items; future work should examine the structure using methods designed for binary or ordered categorical data, such as tetrachoric/polychoric correlation matrices, parallel analysis, and categorical confirmatory factor analysis. Third, confirmatory factor analysis was not performed in an independent sample. Fourth, internal consistency was borderline for the total score and poor for some provisional factors, limiting factor-level interpretation. Fifth, the 12-item model was analytically derived from the full 18-item Greek translation, and the removal of six original items may have reduced content coverage and conceptual equivalence with the original QUIPA. Sixth, one retained item on exercise adherence remains conceptually dependent on a removed stem item on exercise prescription, so the current evidence supports a retained-item scoring model rather than a fully operational stand-alone questionnaire. Seventh, because QUIPA uses a 12-week recall period and participants continued to receive physiotherapy during the 7-day retest interval, changes in reported care-process indicators may partly reflect additional care delivered during that interval rather than measurement stability alone. Eighth, ceiling effects were observed for three retained items, suggesting limited discriminatory capacity for some indicators in this sample. Ninth, convergent validity was not assessed. Tenth, participants were recruited from 10 private physiotherapy clinics, and care processes may have clustered by clinic or therapist; the sample size was insufficient for multilevel modelling, so this potential clustering effect could not be evaluated. Finally, the sample was restricted to adults aged 45–65 years receiving care in private physiotherapy clinics in Attica, limiting generalizability to older adults, rural populations, public-sector services, and the broader Greek osteoarthritis population.

From a clinical perspective, the 12-item Greek QUIPA model may provide a practical descriptive tool for identifying whether patients report receiving key components of evidence-based physiotherapy care, such as education, exercise guidance, self-management support, and individualized management. However, its current application should be limited to settings similar to those examined in this study until additional validation is available.

Future studies should validate the retained Greek 12-item model in an independent sample using methods appropriate for ordered categorical items. Parallel analysis and categorical EFA should be used to examine factor retention, and categorical confirmatory factor analysis should test whether the provisional three-factor structure is replicable. A correlated three-factor model should be tested against a one-factor alternative. Multi-group confirmatory factor analysis should then assess measurement invariance across sex and, importantly, private- versus public-sector settings. Future larger studies should also consider clustering by clinic or therapist. Convergent validity should be examined using a priori hypotheses and comparator instruments that assess related constructs, such as osteoarthritis care-quality measures and shared decision-making/self-management support instruments. Responsiveness should be evaluated longitudinally using predefined hypotheses about change scores and, where possible, an external anchor.

## 5. Conclusions

The present study provides preliminary validity and reliability evidence for a derived 12-item Greek QUIPA short-form model in adults with hip and/or knee osteoarthritis receiving physiotherapy in private clinics in Attica. The model may be useful for descriptive evaluation of whether patients report receiving key evidence-based components of physiotherapy care. However, because the factor structure was obtained under marginal sampling adequacy, several retained items showed low communalities, internal consistency was modest, item-level reliability was affected by categorical and ceiling-response patterns, and the short form was derived from the full 18-item questionnaire, the findings should be interpreted cautiously. At present, the Greek QUIPA is best used as an overall proportion-positive process-of-care indicator rather than as a definitive replication of the original QUIPA subscales or as a fully operational stand-alone short form. Independent confirmatory factor analysis, categorical-data validation, clearer stand-alone short-form specification, convergent validity testing, and validation in more diverse public and community settings are required.

## Figures and Tables

**Table 1:** Demographic and clinical characteristics of the study participants.

Mean ± SD or *n* (%)	Variable
57.37 ± 6.68	Age (years)
82.43 ± 14.84	Weight (kg)
1.68 ± 0.10	Height (m)
29.13 ± 5.25	Body Mass Index (kg/m^2^)
3.67 ± 2.57	Disease Duration (years)
60 (66.7)	Female
30 (33.3)	Male
29 (32.2)	Hip only
53 (58.9)	Knee only
8 (8.9)	Hip & Knee

**Table 2:** Pattern matrix of factor loadings and communalities for the retained items.

Item	Factor 1	Factor 2	Factor 3	Communality
Q1			0.474	0.296
Q4	0.405			0.341
Q7		0.707		0.419
Q8		0.406		0.348
Q9			0.685	0.448
Q11			0.588	0.384
Q12		0.557		0.321
Q13a	0.467			0.269
Q13b	0.496			0.380
Q14	0.741			0.378
Q15	0.593			0.358
Q16			0.444	0.349

**Table 3:** Factor structure and internal consistency of the 12-item three-factor QUIPA model.

Factor (Subscale)	Items	Cronbach’s α
1st Referrals—Guidance & Education	(Q4, Q13a, Q13b, Q14, Q15)	0.662
2nd Information—Management & Adherence	(Q7, Q8, Q12)	0.424
3rd Assessment—Individualization of Physiotherapy Intervention	(Q1, Q9, Q11, Q16)	0.598
Total score	Q1, Q4, Q7, Q8, Q9, Q11, Q12, Q13a, Q13b, Q14, Q15, Q16	0.675

**Table 4:** Categorical item-level test–retest reliability and total-score reliability of the retained Greek QUIPA model.

Item	*n*	Observed Agreement (%)	Cohen’s Kappa	Interpretation
Q1	90	97.8	0.656	Substantial
Q4	90	86.7	0.726	Substantial
Q7	90	95.6	0.580	Moderate
Q8	90	94.4	0.590	Moderate
Q9	90	91.1	0.736	Substantial
Q11	90	91.1	0.714	Substantial
Q12	79	79.7	0.581	Moderate
Q13a	90	93.3	0.887	Almost perfect
Q13b	50	94.0	0.880	Almost perfect
Q14	90	86.7	0.756	Substantial
Q15	90	90.0	0.809	Almost perfect
Q16	90	86.7	0.693	Substantial
Total score	90	Not applicable	ICC = 0.829	Good; 95% CI = 0.748–0.896

**Table 5:** Item-level response distributions and floor/ceiling effects for the retained 12-item Greek QUIPA model. Values are *n* (%) unless otherwise stated.

Item	Yes	No	Don’t Remember	Missing/NA	Valid Positive	Effect
Q1	86 (95.6)	4 (4.4)	0 (0.0)	0 (0.0)	95.6	Ceiling
Q4	19 (21.1)	63 (70.0)	8 (8.9)	0 (0.0)	23.2	None
Q7	83 (92.2)	7 (7.8)	0 (0.0)	0 (0.0)	92.2	Ceiling
Q8	81 (90.0)	9 (10.0)	0 (0.0)	0 (0.0)	90.0	Ceiling
Q9	67 (74.4)	23 (25.6)	0 (0.0)	0 (0.0)	74.4	None
Q11	69 (76.7)	21 (23.3)	0 (0.0)	0 (0.0)	76.7	None
Q12	49 (54.4)	15 (16.7)	15 (16.7)	11 (12.2)	76.6	None
Q13a	46 (51.1)	10 (11.1)	34 (37.8)	0 (0.0)	82.1	None
Q13b	24 (26.7)	27 (30.0)	0 (0.0)	39 (43.3)	47.1	None
Q14	39 (43.3)	44 (48.9)	7 (7.8)	0 (0.0)	47.0	None
Q15	38 (42.2)	48 (53.3)	4 (4.4)	0 (0.0)	44.2	None
Q16	59 (65.6)	27 (30.0)	4 (4.4)	0 (0.0)	68.6	None

## Data Availability

The datasets generated and/or analyzed during the current study, as well as the translated 12-item questionnaire, are available from the corresponding author on reasonable request.

## Supplementary Materials

The following supporting information can be downloaded at: https://www.mdpi.com/article/10.3390/healthcare14162464/s1. Table S1A: Mapping of the original 18 QUIPA items to the derived Greek 12-item model, including item-retention status, original QUIPA domain, factor-loading information, communalities, and rationale for item retention or removal; Table S1B: Baseline response distribution, missingness, and categorical test–retest reliability indices for the original 18 QUIPA items.

## Abbreviations

The following abbreviations are used in this manuscript:

Abbreviation	Full Term
α	Cronbach’s Alpha
BMI	Body Mass Index
EFA	Exploratory Factor Analysis
HOOS	Hip Disability and Osteoarthritis Outcome Score
ICC	Intraclass Correlation Coefficient
KMO	Kaiser–Meyer–Olkin Measure of Sampling Adequacy
KOOS	Knee Injury and Osteoarthritis Outcome Score
OA	Osteoarthritis
PROMs	Patient-Reported Outcome Measures
QUIPA	Quality Indicators for Physiotherapy Management of Hip and Knee Osteoarthritis
SD	Standard Deviation
SPSS	Statistical Package for the Social Sciences
WOMAC	Western Ontario and McMaster Universities Osteoarthritis Index

## References

[1] Hunter D.J., Bierma-Zeinstra S.M.A. (2019). Osteoarthritis. Lancet.

[2] GBD 2021 Osteoarthritis Collaborators (2023). Global, regional, and national burden of osteoarthritis, 1990–2021: A systematic analysis for the Global Burden of Disease Study 2021. Lancet Rheumatol..

[3] Loeser R.F., Collins J.A., Diekman B.O. (2023). Aging and the pathogenesis of osteoarthritis. Nat. Rev. Rheumatol..

[4] Katz J.N., Arant K.R., Loeser R.F. (2021). Diagnosis and treatment of hip and knee osteoarthritis: A review. JAMA.

[5] Kolasinski S.L., Neogi T., Hochberg M.C., Oatis C., Guyatt G., Block J., Callahan L., Copenhaver C., Dodge C., Felson D. (2020). 2019 American College of Rheumatology/Arthritis Foundation guideline for the management of osteoarthritis of the hand, hip, and knee. Arthritis Care Res..

[6] Fernandes L., Hagen K.B., Bijlsma J.W., Andreassen O., Christensen P., Conaghan P., Doherty M., Geenen R., Hammond A., Kjeken I. (2013). EULAR recommendations for the non-pharmacological core management of hip and knee osteoarthritis. Ann. Rheum. Dis..

[7] National Institute for Health and Care Excellence (NICE) (2022). Osteoarthritis in over 16s: Diagnosis and Management (NG226).

[8] Edwards J.J., Khanna M., Jordan K.P., Jordan J.L., Bedson J., Dziedzic K.S. (2015). Quality indicators for the primary care of osteoarthritis: A systematic review. Ann. Rheum. Dis..

[9] Basedow M., Esterman A. (2015). Assessing appropriateness of osteoarthritis care using quality indicators: A systematic review. J. Eval. Clin. Pract..

[10] Campbell S.M., Braspenning J., Hutchinson A., Marshall M. (2002). Research methods used in developing and applying quality indicators in primary care. Qual. Saf. Health Care.

[11] Osterås N., Garratt A., Grotle M., Natvig B., Kjeken I., Kvien T.K., Hagen K.B. (2013). Patient-reported quality of care for osteoarthritis: Development and testing of a quality indicator questionnaire. Arthritis Care Res..

[12] Teo P.L., Hinman R.S., Egerton T., Dziedzic K.S., Kasza J., Bennell K.L. (2020). Patient-reported quality indicators to evaluate physiotherapy care for hip and/or knee osteoarthritis-development and evaluation of the QUIPA tool. BMC Musculoskelet. Disord..

[13] Peter W.F., Hurkmans E.J., van der Wees P.J., Hendriks E.J.M., van Bodegom-Vos L., Vlieland T.P.M.V. (2016). Healthcare quality indicators for physiotherapy management in hip and knee osteoarthritis. Musculoskelet. Care.

[14] Tore N.G., Oskay D., Satış H., Haznedaroglu S. (2022). Cross-cultural adaptation, validity and reliability of the QUIPA tool: Turkish version. Reumatismo.

[15] Beaton D.E., Bombardier C., Guillemin F., Ferraz M.B. (2000). Guidelines for the process of cross-cultural adaptation of self-report measures. Spine.

[16] Boateng G.O., Neilands T.B., Frongillo E.A., Melgar-Quiñonez H.R., Young S.L. (2018). Best practices for developing and validating scales for health, social, and behavioral research: A primer. Front. Public Health.

[17] Costello A.B., Osborne J.W. (2005). Best practices in exploratory factor analysis: Four recommendations for getting the most from your analysis. Pract. Assess. Res. Eval..

[18] Hair J.F., Black W.C., Babin B.J., Anderson R.E. (2019). Multivariate Data Analysis.

[19] World Medical Association (2013). Declaration of Helsinki: Ethical principles for medical research involving human subjects. JAMA.

[20] European Union (2016). Regulation (EU) 2016/679 (General Data Protection Regulation—GDPR). Off. J. Eur. Union.

[21] Nunnally J.C., Bernstein I.H. (1994). Psychometric Theory.

[22] Koo T.K., Li M.Y. (2016). A guideline of selecting and reporting intraclass correlation coefficients for reliability research. J. Chiropr. Med..

[23] Guvendir M.A., Özkan Y.Ö. (2022). Item removal strategies conducted in exploratory factor analysis: A comparative study. Int. J. Assess. Tools Educ..

[24] Dziedzic K., Bierma-Zeinstra S., Vliet Vlieland T., Roos E., Skou S., Hagen K., Osteras N., Pais S., Cordeiro C., Duffy H. (2016). Joint implementation of guidelines for osteoarthritis in Western Europe: JIGSAW-E. Physiotherapy.

[25] Gray-Burrows K.A., Willis T.A., Foy R., Rathfelder M., Bland P., Chin A., Hodgson S., Ibegbuna G., Prestwich G., Samuel K. (2018). Role of patient and public involvement in implementation research: A consensus study. BMJ Qual. Saf..

[26] Bennell K.L., Hinman R.S. (2011). A review of the clinical evidence for exercise in osteoarthritis of the hip and knee. J. Sci. Med. Sport.

