# Supplementary material for: Preliminary Validity and Reliability Evidence for a Derived 12-Item Greek Short-Form Model of the Quality Indicators for Physiotherapy Management of Hip and Knee Osteoarthritis (QUIPA)
Source: Healthcare (Basel). 2026 Aug 10;14(16):2464. doi: 10.3390/healthcare14162464 (PMC13512324; doi:10.3390/healthcare14162464)
Supplement: Supplementary file 1 [file healthcare-14-02464-s001.zip › healthcare-4354065-supplementary.pdf]

Supplementary Table S1. Mapping of the original QUIPA items to the derived Greek 12-item model, item-retention rationale, response distributions, and categorical test–retest reliability

Note. Item content is summarized by theme rather than reproduced as full questionnaire wording.

For retained items, loading and communality values correspond to the final retained 12-item pattern matrix reported in the manuscript. For removed items, loading information is provided from the initial full-pool item inspection only to document the refinement process; removed-item coefficients should not be interpreted as part of the final retained solution.

Scoring follows Yes/(Yes + No) x 100. 'Don't remember', missing, and not applicable responses were excluded from the denominator for valid positive percentages.

Observed agreement and Cohen's kappa were calculated from baseline and 7-day retest responses, treating 'Don't remember' as a response category and excluding missing/not applicable pairs.

Abbreviations: DR, Don't remember; EFA, exploratory factor analysis; NA, not applicable; OA, osteoarthritis; QUIPA, Quality Indicators for Physiotherapy Management of Hip and Knee Osteoarthritis.

Table S1A. Item retention and factor-analytic information

Item	Original QUIPA domain	Item content/theme	Retained	Loading source	Primary loading	Relevant cross-loading(s)	Communality	Decision rationale
Q1	Assessment and Management Planning	Assessment of OA-related problems and needs	Yes	Final retained solution	F3 = 0.474	None >= 0.40	0.296	Retained; conceptually central to assessment, but ceiling effect should be considered.
Q2	Assessment and Management Planning	Comorbidity assessment	No	Initial full-pool inspection	0.168	No salient loading	0.066	Removed; weak/inconsistent contribution to the emerging factor structure.
Q3	Assessment and Management Planning	Psychosocial/depression-related screening	No	Initial full-pool inspection	0.386	F3 = 0.300	0.263	Removed; conceptually ambiguous/inconsistent loading pattern.
Q4	Assessment and Management Planning	Referral or guidance when additional support is indicated	Yes	Final retained solution	F1 = 0.405	None >= 0.40	0.341	Retained; contributed to the provisional referral/guidance/education factor.
Q5	Assessment and Management Planning	Management/treatment planning	No	Initial full-pool inspection	0.724	F1 = 0.307	0.637	Removed; loading pattern and conceptual fit were not sufficiently coherent in the retained solution.
Q6	Assessment and Management Planning	Review or follow-up timing	No	Initial full-pool inspection	0.317	No salient loading	0.149	Removed; poor temporal stability and weak contribution to the common structure.
Q7	Core Recommended Treatments	Information about OA and related pain	Yes	Final retained solution	F2 = 0.707	None >= 0.40	0.419	Retained; salient loading, but ceiling effect may limit discrimination.
Q8	Core Recommended Treatments	Information about management options / self-management	Yes	Final retained solution	F2 = 0.406	None >= 0.40	0.348	Retained; salient loading, but ceiling effect may limit discrimination.
Q9	Core Recommended Treatments	Discussion of individual needs or treatment preferences	Yes	Final retained solution	F3 = 0.685	None >= 0.40	0.448	Retained; salient loading and conceptually relevant to individualized care.
Q10	Core Recommended Treatments	Specific exercise programme prescription	No	Initial full-pool inspection	0.247	No salient loading	0.105	Removed; weak contribution to the retained structure, but removal creates dependency issues for Q12.
Q11	Core Recommended Treatments	Individualized physiotherapy intervention or treatment discussion	Yes	Final retained solution	F3 = 0.588	None >= 0.40	0.384	Retained; salient loading and conceptually relevant to individualized intervention.
Q12	Core Recommended Treatments	Discussion/review of exercise adherence	Yes	Final retained solution	F2 = 0.557	None >= 0.40	0.321	Retained; conceptually relevant, but interpretation depends on the original Q10 skip logic.
Q13a	Core Recommended Treatments	Education about benefits of weight loss	Yes	Final retained solution	F1 = 0.467	None >= 0.40	0.269	Retained; conceptually relevant, but high 'Don't remember' responses require caution.
Q13b	Core Recommended Treatments	Weight-loss strategies/advice	Yes	Final retained solution	F1 = 0.496	None >= 0.40	0.380	Retained; conceptually relevant, but high missing/NA due to conditional logic requires caution.
Q14	Adjunctive Treatments	Walking aid-related advice or support	Yes	Final retained solution	F1 = 0.741	None >= 0.40	0.378	Retained; strongest loading in provisional Factor 1.
Q15	Adjunctive Treatments	Appliances/aids or adjunctive support	Yes	Final retained solution	F1 = 0.593	None >= 0.40	0.358	Retained; salient loading and conceptually relevant adjunctive support item.
Q16	Adjunctive Treatments	Work-related or individualized functional advice	Yes	Final retained solution	F3 = 0.444	None >= 0.40	0.349	Retained; salient loading and conceptually relevant individualized functional/work advice.
Q17	Adjunctive Treatments	Footwear advice	No	Initial full-pool inspection	0.104	No salient loading	0.012	Removed; limited contribution to the common structure and reduced conceptual coherence.

Table S1B. Baseline response distribution and categorical test–retest reliability

Item	Valid positive %	Don't remember n (%)	Missing/NA n (%)	Observed agreement %	Cohen's kappa	Reliability N
Q1	95.6	0 (0.0)	0 (0.0)	97.8	0.656	90
Q2	95.6	0 (0.0)	0 (0.0)	97.8	0.738	90
Q3	70.0	0 (0.0)	0 (0.0)	92.2	0.800	90
Q4	23.2	8 (8.9)	0 (0.0)	86.7	0.726	90
Q5	77.8	0 (0.0)	0 (0.0)	85.6	0.456	90
Q6	96.7	0 (0.0)	0 (0.0)	95.6	-0.017	90
Q7	92.2	0 (0.0)	0 (0.0)	95.6	0.580	90

Item	Valid positive %	Don't remember n (%)	Missing/NA n (%)	Observed agreement %	Cohen's kappa	Reliability N
Q8	90.0	0 (0.0)	0 (0.0)	94.4	0.590	90
Q9	74.4	0 (0.0)	0 (0.0)	91.1	0.736	90
Q10	83.1	6 (6.7)	1 (1.1)	85.4	0.468	89
Q11	76.7	0 (0.0)	0 (0.0)	91.1	0.714	90
Q12	76.6	15 (16.7)	11 (12.2)	79.7	0.581	79
Q13a	82.1	34 (37.8)	0 (0.0)	93.3	0.887	90
Q13b	47.1	0 (0.0)	39 (43.3)	94.0	0.880	50
Q14	47.0	7 (7.8)	0 (0.0)	86.7	0.756	90
Q15	44.2	4 (4.4)	0 (0.0)	90.0	0.809	90
Q16	68.6	4 (4.4)	0 (0.0)	86.7	0.693	90
Q17	70.8	1 (1.1)	0 (0.0)	82.2	0.505	90

Interpretive caution. Because Q12 remained conditionally linked to the removed Q10 stem item, and because Q13b was conditionally applicable, reliability and response-distribution results for these items should be interpreted as supporting the retained-item scoring model rather than a fully operational stand-alone short form.
